# Adipose Tissue Stem Cells for Therapy: An Update on the Progress of Isolation, Culture, Storage, and Clinical Application

**DOI:** 10.3390/jcm8070917

**Published:** 2019-06-26

**Authors:** Dinh-Toi Chu, Thuy Nguyen Thi Phuong, Nguyen Le Bao Tien, Dang Khoa Tran, Le Bui Minh, Vo Van Thanh, Pham Gia Anh, Van Huy Pham, Vu Thi Nga

**Affiliations:** 1 Faculty of Biology, Hanoi National University of Education, Hanoi 100000, Vietnam; 2 School of Odonto Stomatology, Hanoi Medical University, Hanoi 100000, Vietnam; 3 Department of Animal Science, College of Agriculture and Life Science, Chonnam National University, Gwangju 61186, Korea; 4 Institute of Orthopaedics and Trauma Surgery, Viet Duc Hospital, Hanoi 100000, Vietnam; 5 Department of Anatomy, University of Medicine Pham Ngoc Thach, Ho Chi Minh City 700000, Vietnam; 6 NTT Hi-tech Institute, Nguyen Tat Thanh University, 300A Nguyen Tat Thanh St., Ward 13, District 4, Ho Chi Minh City 700000, Vietnam; 7 Department of Surgery, Hanoi Medical University, Hanoi 100000, Vietnam; 8 Oncology Department, Viet Duc Hospital, Hanoi 100000, Vietnam; 9 AI Lab, Faculty of Information Technology, Ton Duc Thang University, Ho Chi Minh City 700000, Vietnam; 10 Institute for Research and Development, Duy Tan University, Danang 550000, Vietnam

**Keywords:** adipose tissue stem cells, stem cell therapy, isolation, culture, storage, clinical application

## Abstract

Adipose tissue stem cells (ASCs), known as multipotent stem cells, are most commonly used in the clinical applications in recent years. Adipose tissues (AT) have the advantage in the harvesting, isolation, and expansion of ASCs, especially an abundant amount of stem cells compared to bone marrow. ASCs can be found in stromal vascular fractions (SVF) which are easily obtained from the dissociation of adipose tissue. Both SVFs and culture-expanded ASCs exhibit the stem cell characteristics such as differentiation into multiple cell types, regeneration, and immune regulators. Therefore, SVFs and ASCs have been researched to evaluate the safety and benefits for human use. In fact, the number of clinical trials on ASCs is going to increase by years; however, most trials are in phase I and II, and lack phase III and IV. This systemic review highlights and updates the process of the harvesting, characteristics, isolation, culture, storage, and application of ASCs, as well as provides further directions on the therapeutic use of ASCs.

## 1. Introduction

**Figure 1:**
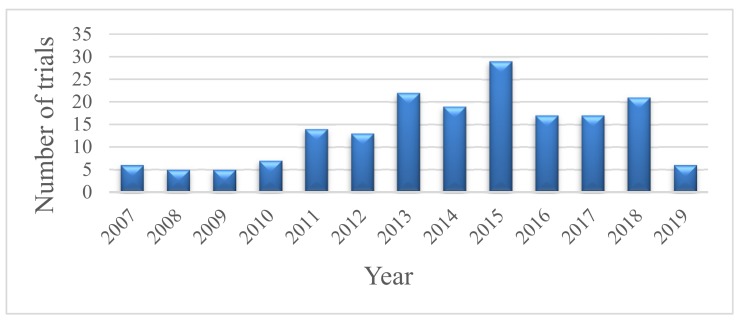
The number of trials on human by year [10].

**Figure 2:**
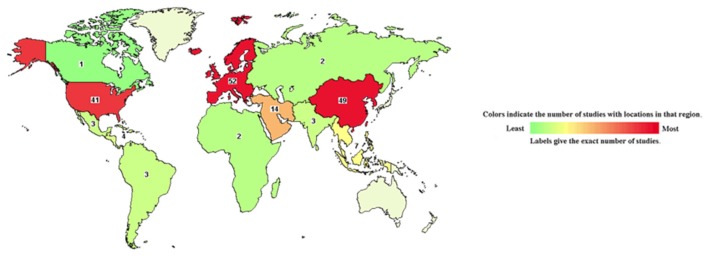
Map of clinical trials on adipose stem cells in the world [11]. East Asia is the highest number of trials, following by Europe, North America, and the United States.

Adipose tissue stem cells (ASCs) are considered as a type of mesenchymal stem cell (MSC) in stromal vascular fractions (SVF) which are isolated from fat tissues enzymatically. Thus, ASCs express the typical surface markers of stem cells and have potentials to differentiate into multiple lineages as MSCs. However, fluorescence-activated cell sorting (FACS) shows the different expressed surface marker profile between MSCs and ASCs. In fact, ASCs are a heterogeneous population consisting of adipose tissue-derived stromal cells and MSCs. In SVFs from adipose tissue and bone marrow, Y. Jang et al. identified six major cell types; while adipose tissue contains a significant number of MSCs and ASCs together with a much lower number of leukocyte than those in the bone marrow. In-vitro, ASCs can be differentiated into osteoblasts, chondroblasts, adipocytes, myocytes, and cardiomyocytes in suitable conditions [1,2,3]. Adipose tissue consists of 100–500 folds higher number of stem cells compared to bone marrow, which makes ASCs an attractive source for human usage. ASCs show therapeutic impacts on angiogenesis, wound healing, and the immune regulatory system. Since the first isolation and classification of ASCs in 2001, the studies about ASCs human trials have been increasing year by year starting from 2007 and reaching its peak in 2015 with up to 187 clinical trials using adipose stem cells. There are six trials registered in clinicaltrial.gov in the first quarter of 2019 (Figure 1). Most of the studies have been conducted in East Asia, Europe, North America and United States (Table 1) in phase I and phase II (Figure 2) for treatment of skeletal diseases, gastrointestinal diseases, skin diseases, nervous disorders, autoimmune diseases, diabetes mellitus, lung and heart diseases. In general, ASCs can be isolated from the collected adipose tissues in patients and directly injected into the wounds, bloodstream, or encapsulated in biomaterials and implanted in the wounds. Many investigations showed ASCs can increase the healing rate and decrease healing time both in-vitro and in-vivo [4,5,6]. ASCs can directly differentiate into specific cell lineages such as keratinocytes, fibroblast-like cells, and endothelial cells, together with the release of growth factors and cytokines, all that promote angiogenesis, development, migration of fibroblasts, and production of fibronectin and collagen. These results are consistent in 14 clinical trials data (in clinicaltrial.gov datasheet). They showed improvement of healing in chronic ulcers and in the reduction of pain. However, further studies are needed to accurate the role of ASCs in cancer therapy [7]. Besides, ASCs are also used in many types of autoimmune diseases such as multiple sclerosis, rheumatoid arthritis, and diabetes mellitus [8]. ASCs contribute to maintaining the balance of the immune system by regulation of T-cell as well as IL-10 secretion and activity. The ASCs encapsulated in biomaterials used for plastic surgeries are considered prevalent in East Asian countries. This system promotes wound healing process and reduces the possibility of scar formation or old scar re-emergence upon the body surface. However, is difficult to control the dosage and safety of ASCs in plastic surgery. Residue collagenase can be harmful to skin or tissue regeneration. It is necessary to determine the effective dose, route, and safety for each indication associated with skin surface or skin conditions in further studies [9].

Despite being discovered only two decades ago, stem cell therapy using ASCs has been developed as regenerative medicine in a series of serious diseases [13,14,15]. Besides, ASCs therapy has been evaluated for advantages and disadvantages in both research and clinical applications. Most authors agreed that ASCs have strong multipotent potential differentiation—easy to harvest and isolate; large and various origins; varying in application method; and high anti-inflammation, immune regulating and angiogenic effects compared to other stem cells and compared to other clinical therapies [8,16]. Most of the investigations considered ASCs transplantation as a safe technique, whereas using SVFs is safer than ASCs [17,18]. Additionally, the side effects of stem cell therapy are regularly originated from the expansion steps which can cause DNA damage linked to senescence and transformation in long-term cultivation [17,19]. ASCs can be isolated from patient adipose tissue in the laboratory and injected back into those patients directly. The quality of ASCs depends on the purifying method and storage condition [19,20]. The cells must be carefully evaluated based on stability, toxicity, contaminations, and senescence in culture [20]. A prospective study in 1524 patients using SVFs was performed in United States from 2011 to 2016. The data showed the safety and improvement outcomes by SVFs [21]. Moreover, preclinical trials have been conducted to evaluate the safety of using human ASCs on animals. In rabbit, there were no systemic side effects by ASCs transplantation to treat osteoarthritis [22]. According to the update data in clinicaltrial.gov until April 2019, there are, in total, 103 trials using ASCs or SVF in early phase I and phase I. The completed trials have shown the safety and positive results of ASC therapies in human [23,24]. Recently, a safety test using adipose tissue MSCs has been done to treat cerebral palsy in a pediatric patient. The patient significantly improved in overall health over a 12-month trial [24].

As with other stem cell therapy, some ASC trials are in terminated or suspended status. According to clinicaltrial.gov, there are 7 terminated trials—NCT01623453, NCT01314092, NCT01011686 (South Korea), NCT02326935 (Cayman Island), NCT02741362 (United State), NCT01378390 (Europe), and NCT01532076 (Switzerland). The failure may be related to insufficient financial support [25]. The effects of ASC therapy vary on the type of cell and transplantation route. Thus, it is necessary to further evaluate large-scale ASC transplantation studies in animal and human trials.

## 2. Characteristics of ASCs

Adipose tissue is regarded as an abundant source of adult stem cells and easy to access in the human body. White adipose tissues (WAT), the main storage of energy, contain huge numbers of ASCs compared to brown adipose tissues (BAT), Besides, ASCs isolated from WAT and BAT showed unlike characteristics in the differentiation ability. In clinical applications, ASCs have mostly been isolated from WAT in subcutaneous depots. It is important to develop and optimize ASCs-related technologies such as the harvesting, isolation, and storage of these cells, which are suitable for clinical application. Generally, ASCs can be obtained in SVFs after removing no-adhesion-plastic cells after 24 h culture. The cell population will be classified based on specific markers on cell surfaces by flow cytometry. Quality and quantity of ASCs thus depend on the isolation methods. Interestingly, most studies showed ASCs are heterogeneous population characterized by groups of surface markers, containing MSCs, adipose stromal cells, endothelial progenitors, pericytes and hematopoietic cells. Compared to bone marrow, the number of SVF cells, or even MSCs and ASCs in adipose tissue are 4–6 folds higher, 4.28% for MSCs and 32% for adipose stromal cells respectively in SVFs [16]. The selected markers for classification of ASCs population may vary by investigations [26,27]. ASCs can express MSC marker CD90 and other markers such as CD34, CD73, and CD105 [28], which is consistent with minimal criteria for defining MSCs by the International Society for Cellular Therapy (ISCT) and the International Fat Applied Technology Society (IFATS) [29,30]. According to IFATS, original ASCs can be defined by positive marker CD34 and negative markers CD45, CD235a and CD31; while cultured ASCs expressed CD73, CD90, CD105, CD44 and were negative with CD45 and CD31. The authors pointed out that CD36+/CD106− markers can be used for distinction with bone marrow stem cells [29]. The morphology and gene expression profile of ASCs are similar to other MSCs derived from bone marrow and umbilical cord. Wolfgang Wagner et al. showed 25 up-regulated genes which overlapped between MSCs from different sources and isolation methods [31]. This suggests using 25 defined genes to classify stem cells among others cell population together with cell surface markers by screening method. In the expansion culture, ASCs can achieve the maximum proliferation in 10% Fetal Bovine Serum (FBS) medium or even a mixture of low FBS and separate growth factor medium [32]. ASCs showed similar morphology and differentiation characteristics to MSCs from bone marrow [33]. ASCs can be differentiated into adipocytes, osteoblasts, chondroblasts, hepatocytes, and neuron cells in different conditions [2,3,26,33,34,35,36,37,38]. In addition, ASCs is also going to be senescence; however, this characteristic is varied by patients [33], and it may be a challenge to ASCs researches.

## 3. Processes in Isolation of ASCs

**Figure 3:**
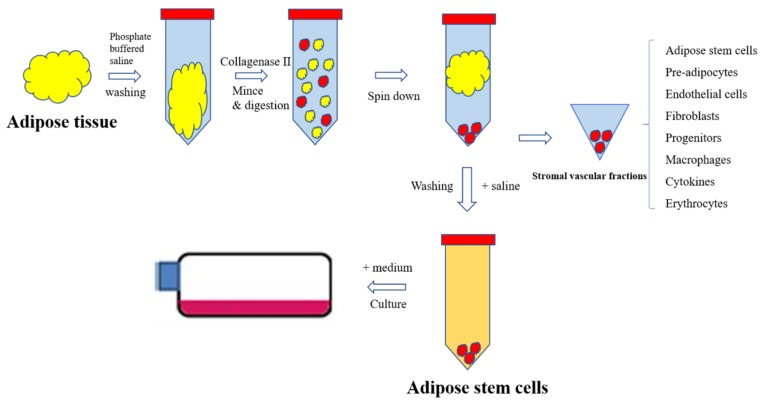
Enzymatic processes in isolation of adipose stem cells.

The isolation processes of ASCs have been developed and optimized for 20 years, which is more suitable for therapeutic application. Zuk et al. first isolated ASCs in 2001 by using collagenase type II to digest adipose tissues and washing with NH4Cl several times which was summarized in Figure 3 [39]. This enzymatic isolation process is most useful and effective for clinical use. In 2017, Raposio et al. developed the standard protocol for isolation of ASCs that showed the maximum in the number of stem cells obtained from liposuction (9.06 × 10^5^ cells per 100mL of adipose tissue) with 99% cell vitality [40]. This process was performed in a closed circulation with minimum contamination and minimal time consumption, that guarantees safety and efficiency of stem cells for clinical uses. Besides the enzymatic method, adipose tissue can be digested by the non-enzymatic method [27,41]. The non-enzymatic method is proposed as an economical method and effective for fat grafting in skin diseases [41]. However, in term of the number of collected ASCs, the enzymatic isolation method is more effective than the non-enzymatic process, 25.9% and 5%, respectively [27].

ASCs are isolated from SVFs, a group of many cell types. SVF cells have different characteristics as well as surface markers. Classification ASCs may be based on specific markers, which were screened by flow cytometry. Moreover, Zuk and his groups demonstrated that it is impossible to separate SVFs population by single marker [39]. The standard specific surface markers of ASCs are still developing. Researchers have been used different surface markers to classify SVFs. However, they totally agree that ASCs express MSCs markers such as CD90 [16,27,42,43]. ASCs positively express CD34^+^, CD90^+^, and negatively express CD31^−^, CD34^+/−^, CD45^−^, CD105^−^, and CD146^−^ markers [33], while other authors investigated different positively expressed markers such as CD73^+^ in ASCs [27]. ASCs also share the same surface markers to endothelial progenitors and pericytes [33]. Pham et al. also reported the list of markers expressed by ASCs, which are varied by studies [26]. Expression of those markers is dependent on isolation method, time incubation, and culture condition, which are associated with a different marker profile in primary and cultured cells [44]. However, the isolation method can be done by the closed and automatic system which reduces clinical intervention and prevents any contamination [45,46]. In 2013, Joel A. Aronowitz and Joshua D.I. Ellenhorn evaluated 4 semi-closed isolation systems. The authors reported the Celution System with automatic procedure can obtain the highest number of viable cells, ASCs with the lowest number of residual enzymes compared to manual systems as Multi Station, Lipokit, and Cha-Station [46]. In 2017, Jonathan Rodriguez et al. continued to compare the quality, morphology, proliferation, and differentiation of obtained ASCs from 3 closed devices (GID-SVF1, Stem.Pras, and Puregraft). The authors emphasized the equivalent of ASCs quality among 3 systems, the possibility of practical application as well as GMP facilities [46]. The current ASCs or SVFs isolation systems from adipose tissues are summarized in the Table 2. Besides, the closed and sterilized kits are also available for research use, such as SynGenX-1000, SynGenX-2000, Sepax-2, and StromaCell.

## 4. Processes in the Culture of ASCs

It is clear that both freshly isolated SVF cells and cultured ASCs are currently used in clinical applications because of their premium characteristics in regulatory requirements, facilities, cost, dose, and time consumption [47,48,49]. Expansion culture of ASCs amplifies and produces a homogeneous cell population that eliminates the variability of the donor factors. However, this process generates more chances for contamination as well as losing stem cell characteristics during long-term culture [47,50,51]. Thus, the culture of ASCs should be carefully evaluated for quality and safety prior to clinical use. The expansion culture of ASCs should be satisfied with the good manufacturing practice (GMP) guidelines which are reported and validated by Hamid-Reza Aghayan et al. [52]. The protocol covers the step-by-step method of the collection, isolation, cultivation, and storage of ASCs. Hamid-Reza Aghayan et al. also mentioned the importance of in-process control (IPC) and quality control of ASCs product. It is useful to standardize the manufacturing process of ASCs and minimize the risks regarding the quality and safety of the product for clinical uses. Phuc Van Pham and his group established some additional guidelines for ASCs manufacturing [53]. By this, the cultured ASCs must be checked for specific surface markers and cell viability, which are related to their phenotypes, self-renewable, and differentiation ability. The authors also demonstrated that hypoxic growth environments are one of the criteria in GMP guideline, which is consistent with the fact that low oxygen can promote growth rates and maintain stem cell functions [50]. Recently, Francesco et al. validated and improved the GMP grade protocol for ASCs isolation, expansion, and storage from SVFs freezing and thawing [54]. DMEM/F-12, α-MEM or DMEM/low glucose media supplement with FBS, growth factors, or cytokines are generally used in traditional ASCs culture [49,53]. Since serum supplement in culture medium can cause allergies related to xenogeneic proteins to patients [17,53], the xeno-free cultures have been developed with the addition of human serum, plasma platelet lysate, or platelet-rich plasma instead of FBS or fetal calf serum (FCS). In early 2018, the large-scale preparation of ASCs using platelet lysate in a closed and automatic system has been developed [55]. The investigation showed 5% human platelet lysate supplied in the system can significantly increase the proliferation rate whereas maintaining the genetic feature of ASCs compared to 10% FBS supplement. This finding can be helpful to prepare ready-to-use allogeneic ASCs for clinical application. Besides, human serum enhances the ASCs proliferation without any differences in morphology or immunostaining profile compared to traditional culture with FBS [53]. Blood-derived products consisted of many growth factors compared to FBS or FCS can be useful to maintain the high proliferation rate, stem cell properties, and differentiation characteristics of ASCs in long-term cultivation [53,56]. Moreover, the combination of platelet lysate and human plasma which are isolated from expired blood can reduce senescence of ASCs during cultivation [56]. Besides, platelet-rich plasma added medium can stimulate the differentiation of ASCs into chondroblasts [57]. Thus, recently, the serum-free medium has been developed commercially [17]. STK2 medium is a chemically defined medium, which obtains a higher proliferation rate, induces stem cell surface markers as well as reduces cell senescence compared to FBS [58]. The free serum and free xenogeneic-protein medium still stimulate the proliferation of ASCs with the expression of entirely stem cell morphology, surface markers, as well as differentiation ability [59]. On the other hand, a research group succeeded in the scale-up of ASCs production (35L scale) in low-serum conditions [60]. However, there is lack of safety evaluation and the serum-free mediums are mainly applied for research tests, not for clinical trials.

Currently, the instruments using in ASCs production are available in the market with a variety of scales from 0.5 L to 200 L of materials. Small and pilot scale systems can be suitable for autologous ASCs, whereas the large-scale systems for allogeneic ASCs manufacturing. The expansion systems are dynamic bioreactors operated with microcarriers. Dufey et al. and Lipsitz et al. succeeded in producing MSCs preparation which satisfied GMP requirements [61,62]. Lawson et al. co-cultured ASCs and MSCs in the 50 L scale devices and reached the highest proliferation rate [63]. Interestingly, different microcarriers and coating materials can cause changes in the cellular phenotype and gene expression profile. Therefore, it is necessary to develop and validate GMP-grade microcarriers in the system [64].

In conclusion, developed medium with optimal components together with the automatic and closed instruments are useful for expansion of ASCs used in clinical transplantation. Xeno-free medium or xeno-free/serum-free medium is commercial that is the advance of ASCs culture.

## 5. Processes in the Differentiation of ASCs

Adipose-derived stem cells are widely accepted the differentiation capability into the multilineage cells, including adipocyte, osteocyte, chondrocyte, neuron, and other cell types [6]. The cell-surface markers also show the differentiation potency of ASCs. Positive marker CD146 ASCs are more favorable to differentiate into adipocytes than negative CD146 cells [65]. In addition, the differentiation condition generally drives the differentiation capability of ASCs. For instance, supplemented IBMX and insulin promote ASCs into the adipogenic lineage, while β-glycerol phosphate, vitamin, and amino acid stimulate to form osteogenic lineage completely [6,26]. Insulin-like growth factor I is reported as an inducer of ASCs differentiation into hepatocytes [66]. The mechanism of ASCs differentiation has been investigated. Osteogenic differentiation is reported to be link to several pathways which consists of bone morphogenetic proteins, Wnt/β-catenin, fibroblast growth factor, PKA, and ERK1/2 pathways [67,68]. Zinc sulfate regulating bone formation can promote osteogenic differentiation of ASCs in rat [69]. Neural differentiation requires the induction of βFGF and forskolin into several cell types [70,71]. Sujeong Jang et al. investigated that neural induction is associated with Wnt5a/JNK pathway, and not Wnt/β-catenin pathway [70]. In-vivo, after implantation, most ASCs die because of the stressful environment which is related to more complicated mechanism than in-vitro studies [11]. The scaffolds usually used in in-vivo studies supported the proliferation and differentiation of implanted cells via their chemical compositions and physical properties [69]. Collagen can be used to efficiently support the differentiation of ASCs into various lineages than alginate. Both silica nanoparticles, hyaluronic acid induce ASCs proliferation in the implanted site, while other synthetic polymers can stimulate ASCs immobilization, development and differentiation after transplantation. Different scaffolds and tissue engineering methods can be applied to suitable implantation purposes. PDM combined XLHA scaffolds usually apply in the adipogenic differentiation; PLA-PPᵧ scaffolds use for osteogenic differentiation and HA scaffolds for chondrogenic differentiation [69]. All those factors have been developed and optimized to be appropriate for applying ASCs for therapeutic purposes.

## 6. Processes in the Storage of ASCs

ASCs can be used in clinical as regenerative medicine. The storage condition is important to ensure the quality of ASCs preparation which can affect the outcome of therapy. The long-term, as well as short-term storage conditions of ASCs, have been investigated [72,73,74]. The media and temperature were optimized for enhancement of ASCs viability before isolation and application [72]. According to Wu et al. results, ASCs decreased their proliferation by time preservation. ASCs preparation should be stored in the supplement of 10% human serum or platelet-rich plasma in 0.9% saline solution at 4 °C and used in the first 2 h, no longer than 4 h. This result is consistent with the investigation of Nofianti et al. in 2018 [73]. Physiologic saline showed higher efficiency in cell viability of ASCs compared to DMEM medium. For long-term storage, ASCs can be stored at −80 °C or liquid nitrogen up to 6 months. In those conditions, ASCs can maintain the proliferation and differentiation characteristics in culture. However, ASCs membrane may break in case of rapid freezing. The cell viability of 194 cryopreserved ASCs of different ages in 5 years of experiment were evaluated. In that, there is no correlation between the recovery and volume of collected tissue, time of preservation, and patient age [75]. Moreover, in the recent study, 10-year-old ASCs have been evaluated the viability, cell morphology, and cell-surface markers compared to fresh ASCs and short-term ASCs [74]. The cell viability is similar in long-term and short-term stored ASCs, 78% and 79%, respectively. However, long-term storage can affect the differentiation potential. The results showed the highest osteogenic genes expressed in short-term ASCs compared to a fresh group, while lost expression in long-term groups. Adipogenic genes are not affected by time preservation. Those results suggest using storage banks of harvested adipose tissue and thawing the estimated amount of tissue to the isolation of ASCs together with an evaluation of the quality of ASCs before implantation into patients. Besides, SVFs aliquots can be stored as the source of ASCs. SVFs should be frozen at the appropriate cell number in the serum supplemented 5% DMSO [54].

## 7. Processes in the Clinical Application of ASCs

Human adipose-derived stem cells are safe to apply to the treatment of various diseases for years [76]. ASCs can be used in regenerative medicine and immunomodulators. ASCs has potential in differentiation into multiple cell lineages, which is useful for regenerative uses. ASCs have been applied in the treatment of cardiac-related diseases and tissue engineering. ASCs therapy enhances myocardial tissue regeneration and improves their functions [77,78,79]. Treatment of ASCs therapy in 4 weeks in pid is a safe and effective method,) resulting in the improvement of left ventricular ejection fraction and reduction of scar volume in the ventricular wall [79]. The StemBell technique divers ASCs to infarcted injuries, which significantly improved cardiac function compared to ASCs treatment only in rat [80]. In addition, ASCs are also seeded in biomaterials as a scaffold and implanted in the soft tissues. ASCs can reduce scar formation and promote the healing process in cutaneous wounds [81]. Besides, electrical stimulation of viability and differentiation of ASCs in scaffolds supports the treatment of bone defect and regeneration of smooth muscle tissue [82,83].

ASCs can regulate the immune systems by anti-inflammatory potential [84]. ASCs treatment is effective for multiple sclerosis, rheumatoid arthritis, and diabetes mellitus [8]. Several randomized controlled trials reported the positive results of using ASCs injections in knee and hip osteoarthritis patients with no side effects [85,86,87,88]. Isolated ASCs have been implanted or injected to osteoarthritis patients. The studies of ASCs application are more focus on the treatment of knee osteoarthritis. Kim et al. first assessed the benefits of ASCs implantation and injection in patients with knee osteoarthritis in 2015 [89,90]. Both investigations showed significant pain relief and the improvement of cartilage lesions in MRI images; however, ASCs implantation showed higher improvement index value than ASCs injection group after a 1-year follow-up trial [90]. The efficiency of ASCs injections in osteoarthritis did not associate with the dose of treatment. Even a low number of ASCs can greatly improve the symptoms of the disease in the knee [91]. Recently, other trials have continued to be conduct on humans to determine the efficiency and safety of ASCs in osteoarthritis. Jones et al. reported the guideline to evaluate the treatment of ASCs in osteoarthritis, consisting of recruitment, ASCs preparation, and injection, analysis the results, and follow-up [54]. Last year, a 3-year follow up study also confirmed the potential benefits and safety of using ASCs as regenerative medicine in the treatment of osteoarthritis [92]. Supported to that, two different groups in Korea and Italy reported similar improvement results in pain and MRI image and other improvement index factors in the participants [87,88]. In the trials, Lee et al. used cultured ASCs injection, while Roato et al. used the uncultured ASCs to treat the knee of patients. Thus, it is clear that the efficiency of ASCs is not dependent on the dose and form of cells.

Additionally, using ASCs has shown promising results on the treatment of neurodegenerative diseases, including Alzheimer’s disease (AD), Parkinson’s disease (PD), intervertebral disc, amyotrophic lateral sclerosis (ALS), multiple system atrophy [93], Parkinson and traumatic brain injury (TBI) [90,91,92,93,94]. However, the efficiency of ASCs therapy in those neural related diseases is still being investigated. Most investigations were performed in mouse or rat model and were limited in size and selection criteria participants, dosage, and treatment method of ASCs [90,91,92,94,95,96]. Intravenous injection of expanded ASCs ensured high safety rates, better viability and motor activity in ALS mouse [97,98]. In Parkinson disease mouse model, transplanted ASCs recovered the mitochondrial functions, that showed the potential therapeutic of ASCs in the treatment of Parkinson disease [94]. Co-treatment with butylidenephthalide, an extract from a traditional Chinese plant, ASCs therapy also showed the improvement of motor abilities in the experiential model [99]. Currently, in a human trial, the safety test of those ASCs applications has been investigating. The intrathecal treatment of ASCs was safe in the single and double dose in 27 patients with ALS [93]. Two phase I trials of ASCs treatment by injection was also reported in clinicaltrials.gov with positive results. However, there are only two trials of ASCs implantation in Parkinson patients from 2014, but they have not yet recruited participants. There is still need for more studies to evaluate the efficiency of ASCs in those neural related diseases. For five years recently, most ASCs therapy has been studied in animal [100]. In human, ASCs showed the ability to promote cell proliferation and cell viability of nucleus pulposus cells in the intervertebral disc [101,102]. SVF, non-insolated ASCs from adipose tissue, can also improve the outcome in degenerative disc disease with no adverse effects [103]. Autologous ASCs transplantation also recovered the slightly neural function of the spinal cord [104]. ASCs transplantation enhanced endogenous neurogenesis and neural functions in AD mice model [84]. A case report in human also showed the positive effects of autologous ASCs administration in moderate AD patients [105]. There are 2 clinical trials which are using autologous ASCs in the treatment of AD (NCT02899091 and NCT029122169). Interestingly, most tests or trials are conducted with non-expanded ASCs. In fact, the immune-regulatory potential and expressed surface markers in ASCs are depended on the culture conditions, expansion time, and supplements which are associated with adverse effects in AD treatment [106,107].

Besides, ASCs therapy is also intended in the treatment of sport injuries. Stem cells have been researched the benefits in the recovery function of anterior cruciate ligament [108,109], tendon, and elbow injuries [110,111]. Even then, the use of stem cell on sports injuries is still controversial [112]. There is not enough evidence for the safety and efficiency of adipose stem cells therapy in sport.

In conclusion, even though ASCs have been isolated and researched for years, ASCs application is more and more focused as a source of adult stem cells in various diseases. Preclinical and clinical studies of ASCs application were summarized on Table 3. However, the in-vitro experiments or human trials are limited, thus it is necessary to conduct new studies which continue to support the use of ASCs therapy in therapeutic treatment.

## 8. Conclusions

Human adipose stem cells have been researched as regenerative medicine in various diseases with promising results. According to the trials reported in clinicaltrials.gov, the number of trials of ASCs use have increased over the years. With the abundant amount in adipose tissue, ASCs is the most important source of stem cell in adults. Processing of ASCs preparations which consist of harvest, isolation, expansion, and storage have been optimized and standardized to ensure the safety and quality of ASCs for therapeutic use. Additionally, ASCs contained in SVFs can be used directly without an isolation step. In general, ASCs preparation is going to be injected or transplanted in the injury site by using scaffolds. The characteristics of scaffolds might support the adhesion, survival, and proliferation of ASCs, thus contribute to the efficiency of ASCs therapy in use. Currently, ASCs therapy has been applied to the treatment of osteoarthritis, neural, and sporty injuries. However, most investigations have been done in animal models, others in human with limited size sample and condition treatment. Therefore, it is necessary to evaluate the safety and benefits of ASCs therapy in larger participant groups and various conditions of treatment before use.

## 9. Future Directions

Researches on ASCs are about to provide more evidence for the safety and efficiency of using ASCs in clinic application instead of bone marrow stem cells. Processes of ASCs preparation have been refined to greatly contribute to the quality and quantity of ASCs prior to injection or transplantation. Modification of the scaffold components and characteristics drives ASCs location, proliferation, and differentiation into a specific cell line. However, the migration and differentiation of ASCs in-vivo is mostly still uncontrolled. The number of human trials is limited in phase I and phase II; and lacks phase III and IV trials. For the approval of ASCs in clinical use, the further challenge of ASCs can be the standardization of ASCs preparation from different patients and location, the specific healing mechanism of ASCs, effective dose, dose intervals, and appropriate delivery method to load or apply ASCs into patients. That requires the protocols for ASCs processes systematically under quality control. Phase III and phase IV of ASCs in the clinic must be approached with a larger size of participants. Moreover, the combination of ASCs therapy and other methods should be investigated, that can contribute to the promising outcome in both in vitro and in vivo experiments.

## Figures and Tables

**Table 1:** Phase of adipose stem cell trials from 2007 to 2019 [12]

Phase	Trials
**Early Phase I**	6
**Phase I**	97
**Phase I, II**	125
**Phase III**	7
**Phase IV**	2
**Not applicable**	34

**Table 2:** List of current systems for adipose stem cells isolation.

Device	Open/Semi-Closed/Closed	Automatic/Semi-Automatic/Manual	Capacity	Collagenenase Provided (yes/no)	Time (min)	Original Country
Celution	Semi-closed	Automatic	300 g	Yes	90	Cytori Therapeutics, Inc. San Diego, CA, USA
Multi Station	Open	Manual	150 g	No	110	PNC International Co., Ltd. Gyeonggi-do, Korea
Lipokit with 416D	Semi-closed	Manual	100 g	No	110	Medi-Khan Seoul, Korea
Cha-Station	Semi-closed	Semi-automatic	200 g	No	90	PNC International Co., Ltd. Gyeonggi-do, Korea
GID-SVF1 & SVF2	Closed	Manual	300 g	SVF1 (No) SVF2 (Yes)	90	GID Group, Inc. Louisville, Colorado
Stem.pras	Closed	Manual	200 g	No	110	Proteal Spain Mexico
Puregraft 250	Semi-closed	Manual	250 g	No	100	Eurosilicone Zone Industrielle de la Peyrol B.P., France
IcellatorR X	Semi-closed	Semi-automatic	60–100 mL	No	60	Tissue Genesis, Inc. Honolulu, Hawaii
STEM-X	Closed	Automatic	20–800 cc	yes	Not provided	Medikan Co., Ltd. Seoul, Korea
SynGenX-1000	Closed	Semiautomated	250 mL	No	Not provided	SynGen Inc. Sacramento. CA, USA
Sepax-2	Semiclosed	Semiautomated	300 g	No	90	Biosafe Group SA Route Du Petit-Eysins 1 Eysins, Switzerland
StromaCell	Closed	Semiautomated	Not provided	No	Not provided	MicroAire Surgical Instruments, LLC Charlottesville, VA, USA

**Table 3:** Preclinical studies and clinical trials of adipose stem cells (ASCs) applications.

Diseases	Pre-Clinical Studies	Clinical Trials	Routine Treatment	Effect of ASCs Therapy	Autologous or Heterologous	Ref.
Knee osteoarthritis	Yes	-	Intra-articular injections	Improve pain, function and cartilage volume of the knee joint	Autologous	[113]
	-	Phase I	Intra-articular injection	Decrease pain and improve WOMUA index	Autologous	[114]
Degenerative disc disease	-	Yes	Injection	Decrease low back pain	Autologous	NCT02097862
	-	Yes	Intradiscal implantation	Improvement of flexion, pain ratings, VAS, PPI	Stromal vascular fractions	NCT02097862
Hip osteoarthritis	Yes	-	Intra-articular injection	Decrease pain and improve the function subscales	ASCs	[115]
	-	Yes	Percutaneous injections	Regenerate cartilage-like tissue	ASCs	[116]
Cardiac disease	Yes	-	Intracoronary reperfusion	Improve LVEF & reduce infarct area	Autologous ASCs	[117]
Heart failure	-	Phase II	Intramyocardial injection		Heterologous	NCT0267316
Ischemic heart disease	-	Phase I/II	Intramyocardial injection	Increase myocardial perfusion	Autologous	[118]
Ischemic cardiomyopathy	-	Phase I	Intravenous injection	Angiogenic effect	Autologous	NCT00426868
Critical limb ischemia	-	Phase I/II	Intramuscular injection	Angiogenic effect	Autologous	NCT01211028
Chronic myocardial ischemia	-	Phase I/II	Intramyocardial injection	Angiogenic effect	Heterologous	NCT01556022
Ischemic stroke	-	Phase II	Intravenous injection	Angiogenic effect	Heterologous	NCT01678534
Stroke	-	Phase II/III	Intravenous infusion	Angiogenic effect	Heterologous	NCT02849613
Amyotrophic lateral sclerosis	-	Phase I	Intravenous injection	Safety Improvement of ALS function, FVC	ASCs	NCT02492516
	Yes	-	Transplantation	Neuroprotective effects by increasing cytokine & growth factors	ASCs	[119]
	Yes	-	Intravenous injection	Enhance the viability and motor activity	Autologous	[98,99]
Multiple system atrophy	-	Phase I	Intrathecal injections	Safety	Autologous	NCT02315027 *
	-	Phase I/II	intrathecally via lumbar puncture	Safety at high dose	Autologous	[120,121]
Spinal cord injury	-	Phase I/II	Intrathecal transplantation	Recover ASIA and sensory score	Autologous	[104]
Rheumatoid arthritis	yes	-	Infusion	Increase IL10, T-reg production	Autologous	[122]
	yes	-	Co-culture	Inhibit inflammation	Autologous	[123,124]
Type I diabetes mellitus	Yes	-	Intravenous transfusion	Improve glucose & insulin tolerance, increase insulin production	Autologous	[125]
Type 2 Diabetes	Yes	-	Intravenous injection	Increase insulin sensitivity, reduce inflammation and fat mass	Autologous	[126]
Alzheimer’s disease	Yes	-	Transplantation	Enhance neurogenic activity, reduce oxidative stress	Autologous	[127]
	Yes	-	Intravenously or intracerebrally injection	Increase cytokine IL10 & VEGF	Autologous	[128]
Parkinson’s disease	Yes	-	Transplantation	Neurogenesis increase cytokine secretion and brain-derived neurotrophic factor	Autologous	[129]
	Yes	-	Transplantation	Improve motor function & neuroprotective effects	Monolayer-cultured ASCs	[130]
Traumatic brain injury	-	Phase I/II	Injection	Safety Benefits	Autologous	NCT02959294 *

LVEF—left ventricular injection fraction; VAS—Visual analog scale; PPI: present pain intensity; WOMUA index—Western Ontario and McMaster Universities Arthritis Index; ALS—amyotrophic lateral sclerosis; FVC—Forced Vital Capacity; ASIA—American Spinal Injury Association; * Clinical trials are processing.

## References

[1] Rogne M., Chu D.-T., Küntziger T.M., Mylonakou M.-N., Collas P., Tasken K., Parton R.G. (2018). OPA1-anchored PKA phosphorylates perilipin 1 on S522 and S497 in adipocytes differentiated from human adipose stem cells. Mol. Biol. Cell.

[2] Chu D.-T., Tao Y. (2017). Human thermogenic adipocytes: A reflection on types of adipocyte, developmental origin, and potential application. J. Physiol. Biochem..

[3] Chu D.-T., Tao Y., Son L.H., Le D.-H. (2016). Cell source, differentiation, functional stimulation, and potential application of human thermogenic adipocytes in vitro. J. Physiol. Biochem..

[4] Hassan W.U., Greiser U., Wang W. (2014). Role of adipose-derived stem cells in wound healing. Wound Repair Regen..

[5] Shingyochi Y., Orbay H., Mizuno H. (2015). Adipose-derived stem cells for wound repair and regeneration. Expert Opin. Biol. Ther..

[6] Si Z., Wang X., Sun C., Kang Y., Xu J., Wang X., Hui Y. (2019). Adipose-derived stem cells: Sources, potency, and implications for regenerative therapies. Biomed. Pharmacother..

[7] Holm J.S., Toyserkani N.M., Sorensen J.A. (2018). Adipose-derived stem cells for treatment of chronic ulcers: Current status. Stem Cell Res. Ther..

[8] Sabol R.A., Bowles A.C., Côté A., Wise R., Pashos N., Bunnell B.A. (2018). Therapeutic Potential of Adipose Stem Cells. Adv. Exp. Med. Biol..

[9] Kim Y.-J., Jeong J.-H. (2014). Clinical application of adipose stem cells in plastic surgery. J. Korean Med. Sci..

[10] The Number of Trials on Human by Year. https://www.clinicaltrials.gov.

[11] Phase of Adipose Stem Cell Trials from 2007 to 2019. https://www.clinicaltrials.gov.

[12] Map of Clinical Trials on Adipose Stem Cells in the WorLd. https://www.clinicaltrials.gov.

[13] Zhao L., Johnson T., Liu D. (2017). Therapeutic angiogenesis of adipose-derived stem cells for ischemic diseases. Stem Cell Res. Ther..

[14] Gaur M., Dobke M., Lunyak V.V. (2017). Mesenchymal Stem Cells from Adipose Tissue in Clinical Applications for Dermatological Indications and Skin Aging. Int. J. Mol. Sci..

[15] Naderi N., Combellack E.J., Griffin M., Sedaghati T., Javed M., Findlay M.W., Wallace C.G., Mosahebi A., Butler P.E.M., Seifalian A.M. (2017). The regenerative role of adipose-derived stem cells (ADSC) in plastic and reconstructive surgery. Int. Wound J..

[16] Jang Y., Koh Y.G., Choi Y.-J., Kim S.-H., Yoon D.S., Lee M., Lee J.W. (2015). Characterization of adipose tissue-derived stromal vascular fraction for clinical application to cartilage regeneration. In Vitro Cell. Dev. Biol. Anim..

[17] Patrikoski M., Mannerstr B., Mannerström S. (2019). Perspectives for Clinical Translation of Adipose Stromal/Stem Cells. Stem Cells Int..

[18] Bowles A.C., Wise R.M., Gerstein B.Y., Thomas R.C., Ogelman R., Febbo I., Bunnell B.A. (2017). Immunomodulatory Effects of Adipose Stromal Vascular Fraction Cells Promote Alternative Activation Macrophages to Repair Tissue Damage. Stem Cells.

[19] Neri S. (2019). Genetic Stability of Mesenchymal Stromal Cells for Regenerative Medicine Applications: A Fundamental Biosafety Aspect. Int. J. Mol. Sci..

[20] Palumbo P., Lombardi F., Siragusa G., Cifone M.G., Cinque B., Giuliani M. (2018). Methods of Isolation, Characterization and Expansion of Human Adipose-Derived Stem Cells (ASCs): An Overview. Int. J. Mol. Sci..

[21] Berman M., Lander E. (2017). A Prospective Safety Study of Autologous Adipose-Derived Stromal Vascular Fraction Using a Specialized Surgical Processing System. Am. J. Cosmet. Surg..

[22] Riester S.M., Denbeigh J.M., Lin Y., Jones D.L., De Mooij T., Lewallen E.A., Nie H., Paradise C.R., Radel D.J., Dudakovic A. (2017). Safety studies for use of adipose tissue-derived mesenchymal stromal/stem cells in a rabbit model for osteoarthritis to support a phase i clinical trial. Stem Cells Transl. Med..

[23] Lee J.S., Hong J.M., Moon G.J., Lee P.H., Ahn Y.H., Bang O.Y. (2010). A long-term follow-up study of intravenous autologous mesenchymal stem cell transplantation in patients with ischemic stroke. Stem Cells.

[24] Hirano A., Sano M., Urushihata N., Tanemura H., Oki K., Suzaki E. (2018). Assessment of safety and feasibility of human allogeneic adipose-derived mesenchymal stem cells in a pediatric patient. Pediatr. Res..

[25] Trounson A., McDonald C. (2015). Stem cell therapies in clinical trials: Progress and challenges. Cell Stem Cell.

[26] Pham P.V. (2014). Adipose stem cells in the clinic. Biomed. Res. Ther..

[27] Raposio E., Simonacci F., Perrotta R.E. (2017). Adipose-derived stem cells: Comparison between two methods of isolation for clinical applications. Ann. Med. Surg..

[28] Raposio E., Caruana G., Petrella M., Bonomini S., Grieco M.P. (2016). A standardized method of isolating adipose-derived stem cells for clinical applications. Ann. Plast. Surg..

[29] Bourin P., Bunnell B.A., Casteilla L., Dominici M., Katz A.J., March K.L., Redl H., Rubin J.P., Yoshimura K., Gimble J.M. (2013). Stromal cells from the adipose tissue-derived stromal vascular fraction and culture expanded adipose tissue-derived stromal/stem cells: A joint statement of the International Federation for Adipose Therapeutics and Science (IFATS) and the International Society for Cellular Therapy (ISCT). Cytotherapy.

[30] Dominici M.L.B.K., Le Blanc K., Mueller I., Slaper-Cortenbach I., Marini F.C., Krause D.S., Deans R.J., Keating A., Prockop D.J., Horwitz E.M. (2006). Minimal criteria for defining multipotent mesenchymal stromal cells. The International Society for Cellular Therapy position statement. Cytotherapy.

[31] Wagner W., Wein F., Seckinger A., Frankhauser M., Wirkner U., Krause U., Blake J., Schwager C., Eckstein V., Ansorge W. (2005). Comparative characteristics of mesenchymal stem cells from human bone marrow, adipose tissue, and umbilical cord blood. Exp. Hematol..

[32] Roxburgh J., Metcalfe A.D., Martin Y.H. (2016). The effect of medium selection on adipose-derived stem cell expansion and differentiation: Implications for application in regenerative medicine. Cytotechnology.

[33] Legzdina D., Romanauska A., Nikulshin S., Kozlovska T., Berzins U. (2016). Characterization of senescence of culture-expanded human adipose-derived mesenchymal stem cells. Int. J. Stem Cells.

[34] Cheng K.-H., Kuo T.-L., Kuo K.-K., Hsiao C.-C. (2011). Human adipose-derived stem cells: Isolation, characterization and current application in regeneration medicine. Genom. Med. Biomark. Health Sci..

[35] Chu D.-T., Tao Y., Taskén K. (2017). OPA1 in Lipid Metabolism: Function of OPA1 in Lipolysis and Thermogenesis of Adipocytes. Horm. Metab. Res..

[36] Chu D.-T., Tao Y. (2017). A homologous stem cell therapy for obesity and its related metabolic disorders. Med. Hypotheses.

[37] Chu D.-T., Malinowska E., Gawronska-Kozak B., Kozak L.P. (2014). Expression of Adipocyte Biomarkers in a Primary Cell Culture Models Reflects Preweaning Adipobiology. J. Biol. Chem..

[38] Chu D.-T., Gawronska-Kozak B. (2017). Brown and brite adipocytes: Same function, but different origin and response. Biochimie.

[39] Zuk P.A., Zhu M.I.N., Mizuno H., Huang J., Futrell J.W., Katz A.J., Benhaim P., Lorenz H.P., Hedrick M.H. (2001). Multilineage cells from human adipose tissue: Implications for cell-based therapies. Tissue Eng..

[40] Raposio E., Bertozzi N. (2017). Isolation of Ready-to-Use Adipose-Derived Stem Cell (ASC) Pellet for Clinical Applications and a Comparative Overview of Alternate Methods for ASC Isolation. Curr. Protoc. Stem Cell Biol..

[41] Bellei B., Migliano E., Tedesco M., Caputo S., Picardo M. (2017). Maximizing non-enzymatic methods for harvesting adipose-derived stem from lipoaspirate: Technical considerations and clinical implications for regenerative surgery. Sci. Rep..

[42] Raposio E., Caruana G., Bonomini S., Libondi G. (2014). A novel and effective strategy for the isolation of adipose-derived stem cells: Minimally manipulated adipose-derived stem cells for more rapid and safe stem cell therapy. Plast. Reconstr. Surg..

[43] Wankhade U.D., Shen M., Kolhe R., Fulzele S. (2016). Advances in adipose-derived stem cells isolation, characterization, and application in regenerative tissue engineering. Stem Cells Int..

[44] Ong W.K., Tan C.S., Chan K.L., Goesantoso G.G., Chan X.H.D., Chan E., Yin J., Yeo C.R., Khoo C.M., So J.B.Y. (2014). Identification of specific cell-surface markers of adipose-derived stem cells from subcutaneous and visceral fat depots. Stem Cell Rep..

[45] Aronowitz J.A., Ellenhorn J.D.I. (2013). Adipose stromal vascular fraction isolation: A head-to-head comparison of four commercial cell separation systems. Plast. Reconstr. Surg..

[46] Rodriguez J., Pratta A.-S., Abbassi N., Fabre H., Rodriguez F., Debard C., Adobati J., Boucher F., Mallein-Gerin F., Auxenfans C. (2017). Evaluation of three devices for the isolation of the stromal vascular fraction from adipose tissue and for ASC culture: a comparative study. Stem Cells Int..

[47] Lockhart R.A., Aronowitz J.A., Dos-Anjos Vilaboa S. (2017). Use of Freshly Isolated Human Adipose Stromal Cells for Clinical Applications. Aesthet. Surg. J..

[48] Zhou L., Song K., Xu L., Zhao F., Tian H., Zhou C., Xu Z., Ge Y., Wu R., Jia R. (2018). Protective Effects of Uncultured Adipose-Derived Stromal Vascular Fraction on Testicular Injury Induced by Torsion-Detorsion in Rats. Stem Cells Transl. Med..

[49] Mei L., Shen B., Ling P., Liu S., Xue J., Liu F., Shao H., Chen J., Ma A., Liu X. (2017). Culture-expanded allogenic adipose tissue-derived stem cells attenuate cartilage degeneration in an experimental rat osteoarthritis model. PLoS ONE.

[50] Riis S., Zachar V., Boucher S., Vemuri M.C., Pennisi C.P., Fink T. (2015). Critical steps in the isolation and expansion of adipose-derived stem cells for translational therapy. Expert Rev. Mol. Med..

[51] He Q., Ye Z., Zhou Y., Tan W.-S. (2018). Comparative study of mesenchymal stem cells from rat bone marrow and adipose tissue. Turk. J. Biol..

[52] Aghayan H.-R., Goodarzi P., Arjmand B. (2014). GMP-compliant human adipose tissue-derived mesenchymal stem cells for cellular therapy. Stem Cells and Good Manufacturing Practices.

[53] Van Pham P., Vu N.B. (2016). In Vitro expansion of mesenchymal stem cells for clinical use. Prog. Stem Cell.

[54] Agostini F., Rossi F.M., Aldinucci D., Battiston M., Lombardi E., Zanolin S., Massarut S., Parodi P.C., Da Ponte A., Tessitori G. (2018). Improved GMP compliant approach to manipulate lipoaspirates, to cryopreserve stromal vascular fraction, and to expand adipose stem cells in xeno-free media. Stem Cell Res. Ther..

[55] Haack-Sørensen M., Juhl M., Follin B., Harary Søndergaard R., Kirchhoff M., Kastrup J., Ekblond A. (2018). Development of large-scale manufacturing of adipose-derived stromal cells for clinical applications using bioreactors and human platelet lysate. Scan. J. Clin. Lab. Investig..

[56] Phetfong J., Tawonsawatruk T., Seenprachawong K., Srisarin A., Isarankura-Na-Ayudhya C., Supokawej A. (2017). Re-using blood products as an alternative supplement in the optimisation of clinical-grade adipose-derived mesenchymal stem cell culture. Bone Jt. Res..

[57] Van Pham P., Bui K.H.-T., Ngo D.Q., Vu N.B., Truong N.H., Phan N.L.-C., Le D.M., Duong T.D., Nguyen T.D., Le V.T. (2013). Activated platelet-rich plasma improves adipose-derived stem cell transplantation efficiency in injured articular cartilage. Stem Cell Res. Ther..

[58] Lee M.-S., Youn C., Kim J.H., Park B.J., Ahn J., Hong S., Kim Y.-D., Shin Y.K., Park S.G. (2017). Enhanced cell growth of adipocyte-derived mesenchymal stem cells using chemically-defined serum-free media. Int. J. Mol. Sci..

[59] Patrikoski M., Juntunen M., Boucher S., Campbell A., Vemuri M.C., Mannerström B., Miettinen S. (2013). Development of fully defined xeno-free culture system for the preparation and propagation of cell therapy-compliant human adipose stem cells. Stem Cell Res. Ther..

[60] Schirmaier C., Jossen V., Kaiser S.C., Jüngerkes F., Brill S., Safavi-Nab A., Siehoff A., van den Bos C., Eibl D., Eibl R. (2014). Scale-up of adipose tissue-derived mesenchymal stem cell production in stirred single-use bioreactors under low-serum conditions. Eng. Life Sci..

[61] Dufey V., Tacheny A., Art M., Becken U., De Longueville F. (2016). Expansion of human bone marrow-derived mesenchymal stem cells in BioBLU 0.3 c single-use bioreactors. Appl. Note.

[62] Lipsitz Y.Y., Timmins N.E., Zandstra P.W. (2016). Quality cell therapy manufacturing by design. Nat. Biotechnol..

[63] Lawson T., Kehoe D.E., Schnitzler A.C., Rapiejko P.J., Der K.A., Philbrick K., Punreddy S., Rigby S., Smith R., Feng Q. (2017). Process development for expansion of human mesenchymal stromal cells in a 50 L single-use stirred tank bioreactor. Biochem. Eng. J..

[64] Sart S., Agathos S.N., Li Y. (2013). Engineering stem cell fate with biochemical and biomechanical properties of microcarriers. Biotechnol. Prog..

[65] Lauvrud A.T., Kelk P., Wiberg M., Kingham P.J. (2017). Characterization of human adipose tissue-derived stem cells with enhanced angiogenic and adipogenic properties. J. Tissue Eng. Regen. Med..

[66] Shabani Azandaryani Z., Davoodian N., Samiei A., Rouzbehan S. (2019). Insulin-like growth factor-I promotes hepatic differentiation of human adipose tissue-derived stem cells. Cell Biol. Int..

[67] Grottkau B.E., Lin Y. (2013). Osteogenesis of Adipose-Derived Stem Cells. Bone Res..

[68] Fathi E., Farahzadi R. (2017). Enhancement of osteogenic differentiation of rat adipose tissue-derived mesenchymal stem cells by zinc sulphate under electromagnetic field via the PKA, ERK1/2 and Wnt/β-catenin signaling pathways. PLoS ONE.

[69] Dai R., Wang Z., Samanipour R., Koo K.-I., Kim K. (2016). Adipose-derived stem cells for tissue engineering and regenerative medicine applications. Stem Cells Int..

[70] Jang S., Park J.-S., Jeong H.-S. (2015). Neural differentiation of human adipose tissue-derived stem cells involves activation of the Wnt5a/JNK signalling. Stem Cells Int..

[71] Jang S., Cho H.-H., Cho Y.-B., Park J.-S., Jeong H.-S. (2010). Functional neural differentiation of human adipose tissue-derived stem cells using bFGF and forskolin. BMC Cell Biol..

[72] Wu Y.D., Li M., Liao X., Li S.H., Yan J.X., Fan L., She W.L., Song J.X., Liu H.W. (2019). Effects of storage culture media, temperature and duration on human adipose-derived stem cell viability for clinical use. Mol. Med. Rep..

[73] Nofianti C.E., Sari I.N., Marlina N., Pawitan J.A. (2018). Temporary storage solution for adipose derived mesenchymal stem cells. Stem Cell Investig..

[74] Shaik S., Wu X., Gimble J., Devireddy R. (2018). Effects of Decade Long Freezing Storage on Adipose Derived Stem Cells Functionality. Sci. Rep..

[75] Badowski M.S., Muise A., Harris D.T. (2018). Patient use of autologous cryopreserved intact adipose tissue from lipoaspirate. AIMS Cell Tissue Eng..

[76] Lalu M.M., McIntyre L., Pugliese C., Fergusson D., Winston B.W., Marshall J.C., Granton J., Stewart D.J. (2012). Safety of cell therapy with mesenchymal stromal cells (SafeCell): a systematic review and meta-analysis of clinical trials. PLoS ONE.

[77] Joo H.J., Kim J.-H., Hong S.J. (2017). Adipose Tissue-Derived Stem Cells for Myocardial Regeneration. Korean Circ. J..

[78] Bai X., Yan Y., Song Y.-H., Seidensticker M., Rabinovich B., Metzele R., Bankson J.A., Vykoukal D., Alt E. (2009). Both cultured and freshly isolated adipose tissue-derived stem cells enhance cardiac function after acute myocardial infarction. Eur. Heart J..

[79] Haenel A., Ghosn M., Karimi T., Vykoukal J., Kettlun C., Shah D., Dave A., Valderrabano M., Schulz D., Azares A. (2018). Unmodified, autologous adipose-derived regenerative cells improve cardiac function, structure and revascularization in a porcine model of chronic myocardial infarction. bioRxiv.

[80] Woudstra L., Krijnen P.A.J., Bogaards S.J.P., Meinster E., Emmens R.W., Kokhuis T.J.A., Bollen I.A.E., Baltzer H., Baart S.M.T., Parbhudayal R. (2016). Development of a new therapeutic technique to direct stem cells to the infarcted heart using targeted microbubbles: StemBells. Stem Cell Res..

[81] Choi S.-K., Park J.-K., Kim J.-H., Lee K.-M., Kim E., Jeong K.-S., Jeon W.B. (2016). Integrin-binding elastin-like polypeptide as an in situ gelling delivery matrix enhances the therapeutic efficacy of adipose stem cells in healing full-thickness cutaneous wounds. J. Control. Release.

[82] Björninen M., Gilmore K., Pelto J., Seppänen-Kaijansinkko R., Kellomäki M., Miettinen S., Wallace G., Grijpma D., Haimi S. (2017). Electrically stimulated adipose stem cells on polypyrrole-coated scaffolds for smooth muscle tissue engineering. Ann. Biomed. Eng..

[83] Zhang J., Neoh K.G., Kang E.T. (2018). Electrical stimulation of adipose-derived mesenchymal stem cells and endothelial cells co-cultured in a conductive scaffold for potential orthopaedic applications. J. Tissue Eng. Regen. Med..

[84] Park M.-J., Kwok S.-K., Lee S.-H., Kim E.-K., Park S.-H., Cho M.-L. (2015). Adipose tissue-derived mesenchymal stem cells induce expansion of interleukin-10-producing regulatory B cells and ameliorate autoimmunity in a murine model of systemic lupus erythematosus. Cell Transplant..

[85] Dall’Oca C., Breda S., Elena N., Valentini R., Samaila E.M., Magnan B. (2019). Mesenchymal Stem Cells injection in hip osteoarthritis: Preliminary results. Acta Bio-Med. Atenei Parm..

[86] Jones I.A., Wilson M., Togashi R., Han B., Mircheff A.K., Vangsness C.T. (2018). A randomized, controlled study to evaluate the efficacy of intra-articular, autologous adipose tissue injections for the treatment of mild-to-moderate knee osteoarthritis compared to hyaluronic acid: A study protocol. BMC Musculoskelet. Disord..

[87] Lee W.-S., Kim H.J., Kim K.-I., Kim G.B., Jin W. (2019). Intra-Articular Injection of Autologous Adipose Tissue-Derived Mesenchymal Stem Cells for the Treatment of Knee Osteoarthritis: A Phase IIb, Randomized, Placebo-Controlled Clinical Trial. Stem Cells Transl. Med..

[88] Roato I., Belisario D.C., Compagno M., Lena A., Bistolfi A., Maccari L., Mussano F., Genova T., Godio L., Perale G. (2019). Concentrated adipose tissue infusion for the treatment of knee osteoarthritis: Clinical and histological observations. Int. Orthop..

[89] Christopoulos P.F., Msaouel P., Koutsilieris M. (2015). The role of the insulin-like growth factor-1 system in breast cancer. Mol. Cancer.

[90] Kappy N.S., Chang S., Harris W.M., Plastini M., Ortiz T., Zhang P., Hazelton J.P., Carpenter J.P., Brown S.A. (2018). Human adipose-derived stem cell treatment modulates cellular protection in both In Vitro and In Vivo traumatic brain injury models. J. Trauma Acute Care Surg..

[91] Tajiri N., Acosta S.A., Shahaduzzaman M., Ishikawa H., Shinozuka K., Pabon M., Hernandez-Ontiveros D., Kim D.W., Metcalf C., Staples M. (2014). Intravenous transplants of human adipose-derived stem cell protect the brain from traumatic brain injury-induced neurodegeneration and motor and cognitive impairments: Cell graft biodistribution and soluble factors in young and aged rats. J. Neurosci..

[92] Gugliandolo A., Bramanti P., Mazzon E. (2019). Mesenchymal Stem Cells: A Potential Therapeutic Approach for Amyotrophic Lateral Sclerosis?. Stem Cells Int..

[93] Staff N.P., Madigan N.N., Morris J., Jentoft M., Sorenson E.J., Butler G., Gastineau D., Dietz A., Windebank A.J. (2016). Safety of intrathecal autologous adipose-derived mesenchymal stromal cells in patients with ALS. Neurology.

[94] Choi H.S., Kim H.J., Oh J.-H., Park H.-G., Ra J.C., Chang K.-A., Suh Y.-H. (2015). Therapeutic potentials of human adipose-derived stem cells on the mouse model of Parkinson’s disease. Neurobiol. Aging.

[95] Ciervo Y., Ning K., Jun X., Shaw P.J., Mead R.J. (2017). Advances, challenges and future directions for stem cell therapy in amyotrophic lateral sclerosis. Mol. Neurodegener..

[96] Chen B.K., Staff N.P., Knight A.M., Nesbitt J.J., Butler G.W., Padley D.J., Parisi J.E., Dietz A.B., Windebank A.J. (2015). A safety study on intrathecal delivery of autologous mesenchymal stromal cells in rabbits directly supporting Phase I human trials. Transfusion.

[97] Uccelli A., Milanese M., Principato M.C., Morando S., Bonifacino T., Vergani L., Giunti D., Voci A., Carminati E., Giribaldi F. (2012). Intravenous Mesenchymal Stem Cells Improve Survival and Motor Function in Experimental Amyotrophic Lateral Sclerosis. Mol. Med..

[98] Marconi S., Bonaconsa M., Scambi I., Squintani G.M., Rui W., Turano E., Ungaro D., D’agostino S., Barbieri F., Angiari S. (2013). Systemic treatment with adipose-derived mesenchymal stem cells ameliorates clinical and pathological features in the amyotrophic lateral sclerosis murine model. Neuroscience.

[99] Chi K., Fu R.-H., Huang Y.-C., Chen S.-Y., Hsu C.-J., Lin S.-Z., Tu C.-T., Chang L.-H., Wu P.-A., Liu S.-P. (2018). Adipose-Derived Stem Cells Stimulated with *n*-Butylidenephthalide Exhibit Therapeutic Effects in a Mouse Model of Parkinson’s Disease. Cell Transplant..

[100] Ohta Y., Hamaguchi A., Ootaki M., Watanabe M., Takeba Y., Iiri T., Matsumoto N., Takenaga M. (2017). Intravenous infusion of adipose-derived stem/stromal cells improve functional recovery of rats with spinal cord injury. Cytotherapy.

[101] Peroglio M., Douma L.S., Caprez T.S., Janki M., Benneker L.M., Alini M., Grad S. (2017). Intervertebral disc response to stem cell treatment is conditioned by disc state and cell carrier: An ex vivo study. J. Orthop. Transl..

[102] Song K., Gu T., Shuang F., Tang J., Ren D., Qin J., Hou S. (2015). Adipose-derived stem cells improve the viability of nucleus pulposus cells in degenerated intervertebral discs. Mol. Med. Rep..

[103] Comella K., Silbert R., Parlo M. (2017). Effects of the intradiscal implantation of stromal vascular fraction plus platelet rich plasma in patients with degenerative disc disease. J. Transl. Med..

[104] Hur J.W., Cho T.-H., Park D.-H., Lee J.-B., Park J.-Y., Chung Y.-G. (2016). Intrathecal transplantation of autologous adipose-derived mesenchymal stem cells for treating spinal cord injury: A human trial. J. Spinal Cord Med..

[105] Koliakos G. (2015). Treatment with adipose stem cells in a patient with moderate Alzheimer’s disease: Case report. J. Neurorestor..

[106] Kwak K.-A., Lee S.-P., Yang J.-Y., Park Y.-S. (2018). Current Perspectives regarding Stem Cell-Based Therapy for Alzheimer’s Disease. Stem Cells Int..

[107] Wang Z., Peng W., Zhang C., Sheng C., Huang W., Wang Y., Fan R. (2015). Effects of stem cell transplantation on cognitive decline in animal models of Alzheimer’s disease: A systematic review and meta-analysis. Sci. Rep..

[108] Eagan M.J., Zuk P.A., Zhao K.W., Bluth B.E., Brinkmann E.J., Wu B.M., McAllister D.R. (2012). The suitability of human adipose-derived stem cells for the engineering of ligament tissue. J. Tissue Eng. Regen. Med..

[109] Jang K.-M., Lim H.C., Hoon Bae J. (2015). Mesenchymal stem cells for enhancing biologic healing after anterior cruciate ligament injuries. Curr. Stem Cell Res. Ther..

[110] De Aro A.A., Carneiro G.D., Teodoro L.F.R., da Veiga F.C., Ferrucci D.L., Simões G.F., Simões P.W., Alvares L.E., de Oliveira A.L.R., Vicente C.P. (2018). Injured Achilles Tendons Treated with Adipose-Derived Stem Cells Transplantation and GDF-5. Cells.

[111] Lee S.Y., Kwon B., Lee K., Son Y.H., Chung S.G. (2017). Therapeutic mechanisms of human adipose-derived mesenchymal stem cells in a rat tendon injury model. Am. J. Sports Med..

[112] Pas H.I., Moen M.H., Haisma H.J., Winters M. (2017). No evidence for the use of stem cell therapy for tendon disorders: A systematic review. Br. J. Sports Med..

[113] Song Y., Du H., Dai C., Zhang L., Li S., Hunter D.J., Lu L., Bao C. (2018). Human adipose-derived mesenchymal stem cells for osteoarthritis: A pilot study with long-term follow-up and repeated injections. Regen. Med..

[114] Pers Y.-M., Rackwitz L., Ferreira R., Pullig O., Delfour C., Barry F., Sensebe L., Casteilla L., Fleury S., Bourin P. (2016). Adipose mesenchymal stromal cell-based therapy for severe osteoarthritis of the knee: A phase I dose-escalation trial. Stem Cells Transl. Med..

[115] Cuervo B., Rubio M., Sopena J., Dominguez M.J., Vilar J., Morales M., Cugat R., Carrillo M.J. (2014). Hip Osteoarthritis in Dogs: A Randomized Study Using Mesenchymal Stem Cells from Adipose Tissue and Plasma Rich in Growth Factors. Int. J. Mol. Sci..

[116] Pak, J.; Lee, J.H.; Park, K.S.; Lee, S.H. Efficacy of autologous adipose tissue-derived stem cells with extracellular matrix and hyaluronic acid on human hip osteoarthritis. *Biomed. Res.*
**2017**. http://www.biomedres.info/biomedical-research/efficacy-of-autologous-adipose-tissuederived-stem-cells-with-extracellular-matrix-and-hyaluronic-acid-on-human-hip-osteoarthritis.html.

[117] Valina C., Pinkernell K., Song Y.-H., Bai X., Sadat S., Campeau R.J., Le Jemtel T.H., Alt E. (2007). Intracoronary administration of autologous adipose tissue-derived stem cells improves left ventricular function, perfusion, and remodelling after acute myocardial infarction. Eur. Heart J..

[118] Qayyum A.A., Mathiasen A.B., Mygind N.D., Kühl J.T., Jørgensen E., Helqvist S., Elberg J.J., Kofoed K.F., Vejlstrup N.G., Fischer-Nielsen A. (2017). Adipose-Derived Stromal Cells for Treatment of Patients with Chronic Ischemic Heart Disease (MyStromalCell Trial): A Randomized Placebo-Controlled Study. Stem Cells Int..

[119] Kim K.S., Lee H.J., An J., Kim Y.B., Ra J.C., Lim I., Kim S.U. (2014). Transplantation of human adipose tissue-derived stem cells delays clinical onset and prolongs life span in ALS mouse model. Cell Transplant..

[120] Singer W., Dietz A., Zeller A., Gehrking T., Schmelzer J., Sletten D., Gehrking J., Coon E., Sandroni P., Benarroch E. (2017). Intrathecal Administration of Autologous Mesenchymal Stem Cells in Multiple System Atrophy—A Phase I/II Dose-Escalation Trial (S11.002). Neurology.

[121] Singer W., Dietz A., Zeller A., Gehrking T., Schmelzer J., Sletten D., Gehrking J., Coon E., Sandroni P., Benarroch E. (2019). Long-Term Administration of Intrathecal Mesenchymal Stem Cells in Multiple System Atrophy—A Compassionate Use Experience (S18.004). Neurology.

[122] Lopez-Santalla M., Mancheño-Corvo P., Menta R., Lopez-Belmonte J., DelaRosa O., Bueren J.A., Dalemans W., Lombardo E., Garin M.I. (2015). Human Adipose-Derived Mesenchymal Stem Cells Modulate Experimental Autoimmune Arthritis by Modifying Early Adaptive T Cell Responses. Stem Cells.

[123] Baharlou R., Ahmadi-Vasmehjani A., Faraji F., Atashzar M.R., Khoubyari M., Ahi S., Erfanian S., Navabi S.-S. (2017). Human adipose tissue-derived mesenchymal stem cells in rheumatoid arthritis: Regulatory effects on peripheral blood mononuclear cells activation. Int. Immunopharmacol..

[124] Baharlou R., Ahmadi-Vasmehjani A., Rashidi N., Khoubyari M., Sheikh M., Erfanian S. (2018). Immunomodulatory Effects of Human Adipose Tissue-Derived Mesenchymal Stem Cells on T Cell Subsets in Patients with Rheumatoid Arthritis. Iran. J. Allergy Asthma Immunol..

[125] Dang L.T.-T., Bui A.N.-T., Nguyen C.L.-T., Truong N.C., Thi-Van Bui A., Kim N.P., Truong K.D., Van Pham P. (2017). Intravenous Infusion of Human Adipose Tissue-Derived Mesenchymal Stem Cells to Treat Type 1 Diabetic Mellitus in Mice: An Evaluation of Grafted Cell Doses. Stem Cells: Biology and Engineering.

[126] Wang M., Song L., Strange C., Dong X., Wang H. (2018). Therapeutic effects of adipose stem cells from diabetic mice for the treatment of type 2 diabetes. Mol. Ther..

[127] Yan Y., Ma T., Gong K., Ao Q., Zhang X., Gong Y. (2014). Adipose-derived mesenchymal stem cell transplantation promotes adult neurogenesis in the brains of Alzheimer’s disease mice. Neural Regen. Res..

[128] Kim S., Chang K.-A., Kim J.A., Park H.-G., Ra J.C., Kim H.-S., Suh Y.-H. (2012). The Preventive and Therapeutic Effects of Intravenous Human Adipose-Derived Stem Cells in Alzheimer’s Disease Mice. PLoS ONE.

[129] Schwerk A., Altschüler J., Roch M., Gossen M., Winter C., Berg J., Kurtz A., Steiner B. (2015). Human adipose-derived mesenchymal stromal cells increase endogenous neurogenesis in the rat subventricular zone acutely after 6-hydroxydopamine lesioning. Cytotherapy.

[130] Berg J., Roch M., Altschüler J., Winter C., Schwerk A., Kurtz A., Steiner B. (2015). Human adipose-derived mesenchymal stem cells improve motor functions and are neuroprotective in the 6-hydroxydopamine-rat model for Parkinson’s disease when cultured in monolayer cultures but suppress hippocampal neurogenesis and hippocampal memory function when cultured in spheroids. Stem Cell Rev. Rep..

